# New Drugs, Appliances, and Things Medical

**Published:** 1890-11-01

**Authors:** 


### NEW DRUGS, APPLIANCES, AND THINGS MEDICAL.

### THE "SPIRONE" INHALANT.

The Spirone Company have sent us a sample of the above, together with a well-made spray apparatus for its use. Some time back we tried this fluid in cases of spasmodic asthma and also of hay fever. We found that it had considerable effect in relieving the distressing symptoms of these tedious and troublesome complaints. The spray is formed on the correct principles of obtaining the best effect on the air passages by the fluid. That is, it is not a continuous shower of minute drops, but an intermittent one, so that the moment of inhaling is chosen to draw in the spray, and during the act of exhaling the spray is not working. We have no doubt that the remedy will fill a useful place in the list of pulmonary antispasmodics.

TURNING OVER NEW LEAVES.—The authoress of "Modern Malady" sends us a letter, which is too long for insertion, the burden of which is, that the writer of the article "Women and Nerves," in our last issue "shows an utter ignorance of the contents" of Cyril Bennett's book. Having carefully examined the "Modern Malady," we would refer our correspondent especially to the first hundred pages of her work, the contents and purport of which she seems to have forgotten, in proof that, if any confusion has arisen in anybody's mind our reviewer, at any rate, has not suffered from its effects. Authors are sometimes mercifully allowed to forget much that they write, and the present seems to be a case in point.

